# Complete Genome Sequences of New Xenotropic Murine Leukemia Viruses from the Senescence-Accelerated Mouse (SAM): Molecular and Phylogenetic Analyses

**DOI:** 10.1371/journal.pone.0055669

**Published:** 2013-02-05

**Authors:** Yun-Jung Lee, Byung-Hoon Jeong, Eun-Kyoung Choi, Richard I. Carp, Yong-Sun Kim

**Affiliations:** 1 Ilsong Institute of Life Science, Hallym University, Anyang, Gyeonggi-do, Republic of Korea; 2 Department of Microbiology, College of Medicine, Hallym University, Chuncheon, Kangwon-do, Republic of Korea; 3 New York State Institute for Basic Research in Developmental Disabilities, Staten Island, New York, United States of America; College of Medicine, Hallym University, Republic of Korea

## Abstract

Approximately 10% of the mouse genome is constituted by endogenous retroviruses (ERVs), and a number of mouse ERVs remain active. Many copies of endogenous murine leukemia viruses (MuLVs) are detected in the genomes of inbred mouse strains. Some of these MuLVs are transcriptionally active or produce infectious virus particles. Previously, we identified partial *env* sequences of new xenotropic MuLVs (X-MuLVs) from a senescence-accelerated mouse (SAM) strain. In the present study, we investigated and characterized the complete sequences of the X-MuLVs. The complete genomes and open reading frames (ORFs) of two X-MuLVs, designated xmlv15 and xmlv18 (accession nos. HQ154630 and HQ154631, respectively), were molecularly cloned from the genome of the SAM mice. We confirmed that the xmlv15 and xmlv18 sequences are distinct from all known MuLV genomes and are most similar to DG-75 MuLV. Moreover, we found that common strains of laboratory mice carry our newly identified xmlvs. Additionally, the expression levels of xmlv15-related sequences were much higher in C57BL and ICR mice than in the SAM strains without any stimulators. Our findings suggest that a specific group of endogenous MuLVs is constitutively expressed in the brain and that they may participate in normal functions and/or pathogenic conditions.

## Introduction

Approximately 10% of the mouse genome is constituted by chromosomally integrated copies of retrovirus sequences referred to as endogenous retroviruses (ERVs) [1]. These ERV sequences have entered the mouse genome through the spontaneous retroviral infection of germ line cells, and they have been transmitted vertically from one generation to the next [2]. Although a large percentage of ERVs are replication-defective or are suppressed by host defense mechanisms, some ERVs can be expressed and replicated. In particular, as distinct from human ERVs, a number of ERVs are still active in mice [3]. Murine leukemia viruses (MuLVs) account for a small portion of the ERVs found in mice but they are one of the most-investigated classes of these sequences. MuLVs are categorized as class I ERVs, which are most similar to the gammaretrovirus and are morphologically related to type C retroviruses. These sequences include not only replication-defective or suppressed proviruses but also expressed, active ecotropic and xenotropic proviruses [4]. Endogenous MuLVs can be classified into three broad host range groups based on their infectivity: Ecotropic virus (E-MuLV) strains can only replicate in cells of rodent origin; Xenotropic virus (X-MuLV) strains are generally not infectious in mouse cells but can infect cells of other species. Polytropic virus (P-MuLV) strains are produced in mice and can infect mouse cells as well as cells of other species [5]. Although extensive homology is observed between these three groups, the *env* gene is quite polymorphic, and distinct cell surface receptors are therefore used by the ecotropic and xenotropic/polytropic viruses [6]–[12]. E-MuLVs, which are the best investigated category of these sequences, are found at zero to only a few copies, whereas approximately 20–50 copies of xenotropic and polytropic-related sequences are found in the genomes of most inbred and related-wild mice. Among MuLVs, certain active MuLV proviruses encoding infectious virions have been suggested to represent etiological cofactors of spontaneous leukemogenesis [13].

In a previous study, we analyzed the expression of endogenous MuLVs in the brains of accelerated senescence-prone (SAMP8) mice and identified novel 242-bp xenotropic PCR products. The sequences of these products showed closest homology to the xenotropic virus isolates NZB-9-1 and NFS-Th-1 but were not identical to them, presenting different sequences in the hypervariable region (VRA). It has been suggested that there are unidentified xenotropic proviruses in the genomes of SAM stains, developed by selective inbreeding of the AKR/J strain of mice [14]. In the present study, we therefore investigated the complete sequences of the identified X-MuLVs and characterized their expression in mice.

## Materials and Methods

### Animals

Accelerated senescence-resistant (SAMR1) and SAMP8 mice were originally obtained from Dr. Toshio Takeda (Kyoto University, Kyoto, Japan) and have been maintained as inbred strains in the Korea Ginseng and Tobacco Research Institute (Deajun, Korea) and the Ilsong Institute of Life Science. Pathogen-free SAMR1, SAMP8, ICR and C57BL/6J mice and hamsters (Daehan Biolink, Korea) were housed in a clean facility under natural light-dark cycle conditions (12 h each of light and dark). After the animals were sacrificed by asphyxiation with CO_2_, brains were isolated within minutes and stored at −70°C. Laboratory animal experiments were approved by the Hallym Medical Center Intuition Animal Care and Use Committee. The protocol animal handling was accordance with institutional and international guidelines.

### PCR Amplification of Genomic DNA

DNeasy tissue kit (Qiagen, Hilden, Germany) was used in accordance with the supplier’s instruction to extract genomic DNA from brain tissue. Polymerase chain reaction (PCR) was performed with *Pfu-X* DNA polymerase (SolGent, Daejeon, Korea) using specific primers designed to amplify X-MuLV in SAM mice. The PCR condition followed standard application of *Pfu-X* DNA polymerase with an annealing temperature of 60°C. All of the primer sequences used for amplification of X-MuLV and GAPDH are listed in Table 1(written in the 5′→3′ direction). The obtained PCR products were separated on 1% agarose gels and visualized using ethidium bromide staining under UV light.

### Cloning the *Gag-Env* Region

The *gag-env* region of X-MuLV was amplified with the primers designated for the In-Fusion 2.0 Dry-Down PCR Cloning Kit (Clontech, CA, USA). The PCR product corresponding to the *gag-env* region was directly incorporated into a pcDNA3.1 vector using the In-Fusion Kit according to the manufacturer’s instructions. The recombinant plasmids were then transformed into *E. coli* and bacterial colonies were cultured in 3 ml of LB medium containing 50 µg/ml ampicillin. The recombinant plasmids were extracted using the QIAprep Spin Miniprep Kit (Qiagen, Hilden, Germany).

### RT-PCR Analysis

Total RNA was extracted from brain tissue using the TRIzol® reagent (Invitrogen, CA, USA) according to the supplier’s instructions; this RNA was then stored at −70°C. Single-stranded cDNA was synthesized from DNase (Roche, Basel, Switzerland)-treated RNA by reverse transcription, using oligo-dT and AMV reverse transcriptase (Promega, CA, USA). The PCR conditions that were employed were in accordance with standard applications of GoTaq DNA polymerase (Promega, CA, USA) and used an annealing temperature of 55°C. The resulting PCR products were separated on 1% agarose gels and visualized using ethidium bromide staining under UV light.

### Nucleotide Sequencing and Data Analysis

The DNA sequencing was conducted on an ABI 377 automatic sequencer using a Taq dideoxy terminator cycle sequencing kit (ABI, CA, USA). The ABI 377 DNA Sequencer Data Analysis Program and the Sequence Navigator Software package were used to assemble and edit the nucleic acid sequences. The homology of the sequences was compared with the Blastn using flat master-slave with identities from the National Center for Biotechnology Information (NCBI). A neighbor-joining (NJ) phylogenetic tree [15] was constructed using the CLUSTAL X [16] and TREEVIEW 1.6 programs [17]. Sequence divergences were estimated by the two-parameter method [18]. A bootstrap test was applied to estimate the confidence of the branching patterns of the NJ tree [19].

### Accession Numbers

Accession numbers used for analysis were indicated as follows: AKV MuLV (J01998), Friend MuLV (NC_001372), Moloney MuLV (NC_001501), Rauscher MuLV (NC_001819), feline leukemia virus (NC_001940), gibbon ape leukemia virus (NC_001885), koala retrovirus (AF151794), modified polytropic mERV Chromosome 10 (AC166256; nt 15,191–24,235), modified polytropic mERV Chromosome 7 (AC127565; nt 64,278–73,318), modified polytropic mERV Chromosome 12 (AC153658; nt 85,375–94,415), DG-75 (AF221065), MTCR (NC_001702), MuLV MCF 1233 (U13766), MuLV MCF247 (K02727), XMRV VP35 (DQ241301), XMRV 22Rv1-(FN692043, HQ385319), MuLV NCI-417 (AAC97875), MuLV NZB-9–1 (EU035300, K02730), MuLV NFS-Th-1 (K00011), MX33 (M17327), Rmcf (AY999005), xenotropic mERV Chromosome 4 (AL627077; nt 146,320–155,078), xenotropic mERV Chromosome 4 (AL731663; nt 111,633–120,386), xenotropic mERV Chromosome 7 (AC167466; nt 12,452–21,178), xenotropic mERV Chromosome 13 (CT030655; nt 54,685–63,371), xmlv15 (HQ154630), xmlv18 (HQ154631).

## Results

### Genomic Structure of xmlvs

**Figure 1:**
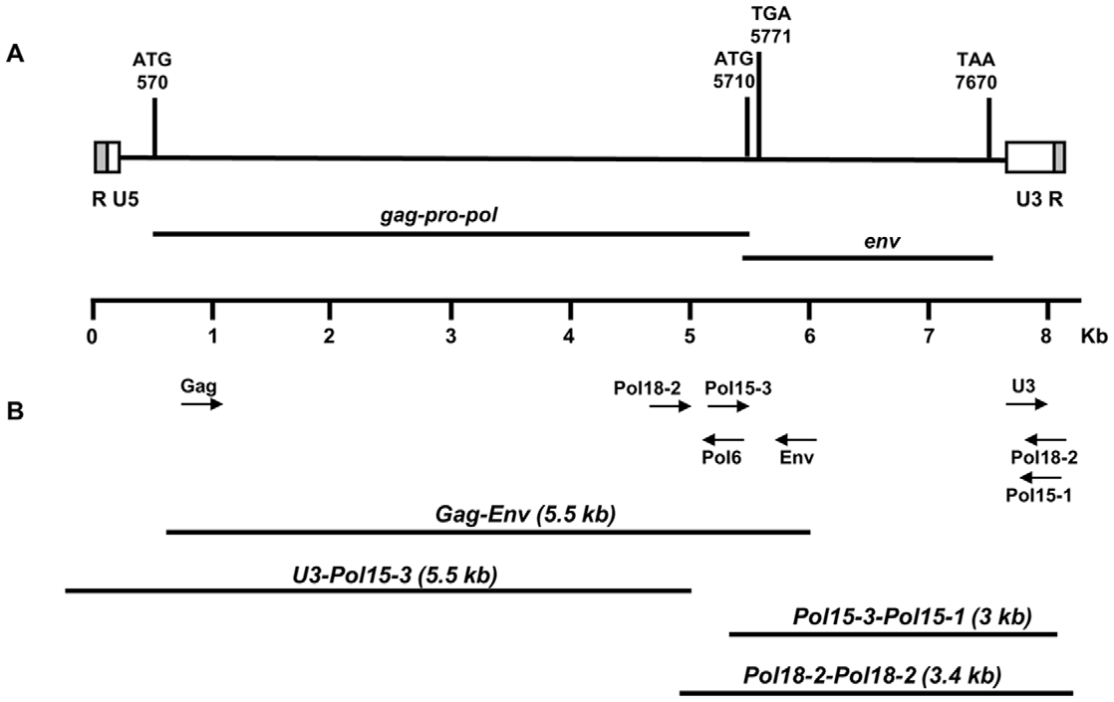
Complete xmlv genome. (A) Schematic map of the 8176 nt genome of xmlv15. The LTR regions (R, U5, U3) are indicated with boxes. Two open reading frames encoding *gag-pro-pol* and *env* polyproteins are predicted. The corresponding start (AUG) and stop codons (UAA) are shown, along with their nucleotide positions. (B) Cloning and sequencing of the xmlvs. The clones obtained by PCR from SAM mouse genomic DNA (black bars) were sequenced. Primers used to amplify individual clones (Table 1) were derived from overlapping xmlv clones (arrows).

To identify new X-MuLV sequences in the genome of the SAM mice, we first amplified a segment from the *gag* to *env* region by PCR using specific primer for *env* sequences that were reported in a previous study. We acquired 4 clones consisting of long segments of the *gag-env* region (approximately 5.5 kb) from the chromosomal DNA of the SAM mice, and 2 different sequences were obtained through sequencing analysis performed in both directions. To investigate the remaining X-MuLV sequences from the SAM mice, we performed PCR amplification of overlapping segments using primers from the clones recovered earlier and aligned the resulting sequences (Fig. 1 and Table 1). We revealed that 8,176 nt complete X-MuLV sequence, xmlv15, and 7,077 nt open reading frame (ORF) sequences, xmlv18, from the SAM mice genome. Xmlv18 is comprised of the *gag, pol* and *env* genes, whereas xmlv15 contained a 5′ and 3′ LTR in addition to the *gag, pol,* and *env* genes. The genomic organization of xmlv15 showed similarity to the organization of mammalian gammaretroviruses (5′-LTR-*gag-pro-pol-env*-3′-LTR). A global pair-wise alignment of xmlv15 and xmlv18 indicated that there were 307 different nucleotides in their ORF regions, resulting in amino acid differences. The two xmlvs share 96% nucleotide and amino acid identities in their predicted ORFs. However the base compositions of their ORFs were the same: 25% A, 25% G, 21% C and 29% C.

**Table 1:** Oligodeoxyribonucleotide primer sequences used for cloning and RT-PCR.

Primer name	Polarity	Sequence (5′ → 3′)
In-fusionGag	Forward	CAGTGTGGTGGAATTCG ACCGTAACCACTCCTTTGA
Pol15-3	Forward	CCAGACAGTGGCCGATTTGT
Pol18-2	Forward	TCCAGAAACTTCCACCCTCC
Pol18-1	Forward	GCCCGATCAGTTTGTGTTTG
U3	Forward	CTGCAGTAACGCCATTTTGCAAGG
In-fusionEnv	Reverse	AACGGGCCCTCTAGATCTTCC GGGAGAGCGGCAACCAAC
Pol15-1	Reverse	CGCCGAGTGTGGAATTTTTA
Pol18-2	Reverse	TGCTGGTTCCGCTTTATCTG
Pol15-3	Reverse	ACAAATCGGCCACTGTCTGG
Pol6	Reverse	CAGCGAGGTTCTAGGTTCTT
Env	Reverse	TCTTCCGGGAGAGCGGCAACCAAC

Through a comparative analysis with other complete sequences of mammalian gammaretroviruses using the BLAST program, we confirmed that the obtained xmlv sequences are distinct from all known MuLV genomes and are most similar to the DG-75 MuLV, 97% and 99% sequence homology with xmlv15 and 18, respectively. The genome sequences of xmlv15 and xmlv18 also have 99% nt identity with xenotropic proviruses on *Mus musculus* Chromosome 4 and Chromosome13, respectively. However, the different nucleotides found in the xmlv15 and xmlv18, 71 nt and 22 nt respectively when they are compared with the most similar xenotrpic proviruses on *M musculus* Chromosome to each xmlv. A phylogenetic analysis constructed with other MuLV strains based on their full genomes also showed that xmlv15 were closely related to X-MuLVs, including mouse xenotropic proviruses, DG-75 MuLV, murine type C retrovirus (MTCR) and xenotropic MuLV-related virus (XMRV) that was isolated from prostate cancer tissue.

### Regulatory Elements of the xmlv15 Genome

Global alignments indicated that xmlv15 LTR is closely related to the LTRs of XMRV-like mouse endogenous retrovirus and DG-75 MuLV, with 97% and 94% identity scores, respectively. Multi-alignment of nucleotide sequences with other well-characterized MuLV strains showed that the xmlv15 LTR was comprised of 433-nt U3, 73-nt repeat (R) and 70-nt U5 regions. The U3 regions of xmlv15 contained well-conserved sequence motifs, such as a CGCTT motif and a TATAA box. Downstream of the U3 regions is the R region made up of 28-nt sequences (GCGCCAGTCCTCCGATAGACTGAGTCGC) that are highly conserved among the mammalian gammaretroviruses and the consensus poly-adenylation signal sequence (AATAAA). The U5 region is followed by a primer binding site (TGGAGGTCCCACCGAGAT), which has a sequence that is complementary to the 3′ end of threonine tRNA.

### Analysis of the xmlv *Gag/Pro/Pol* Polyprotein Region

**Figure 2:**
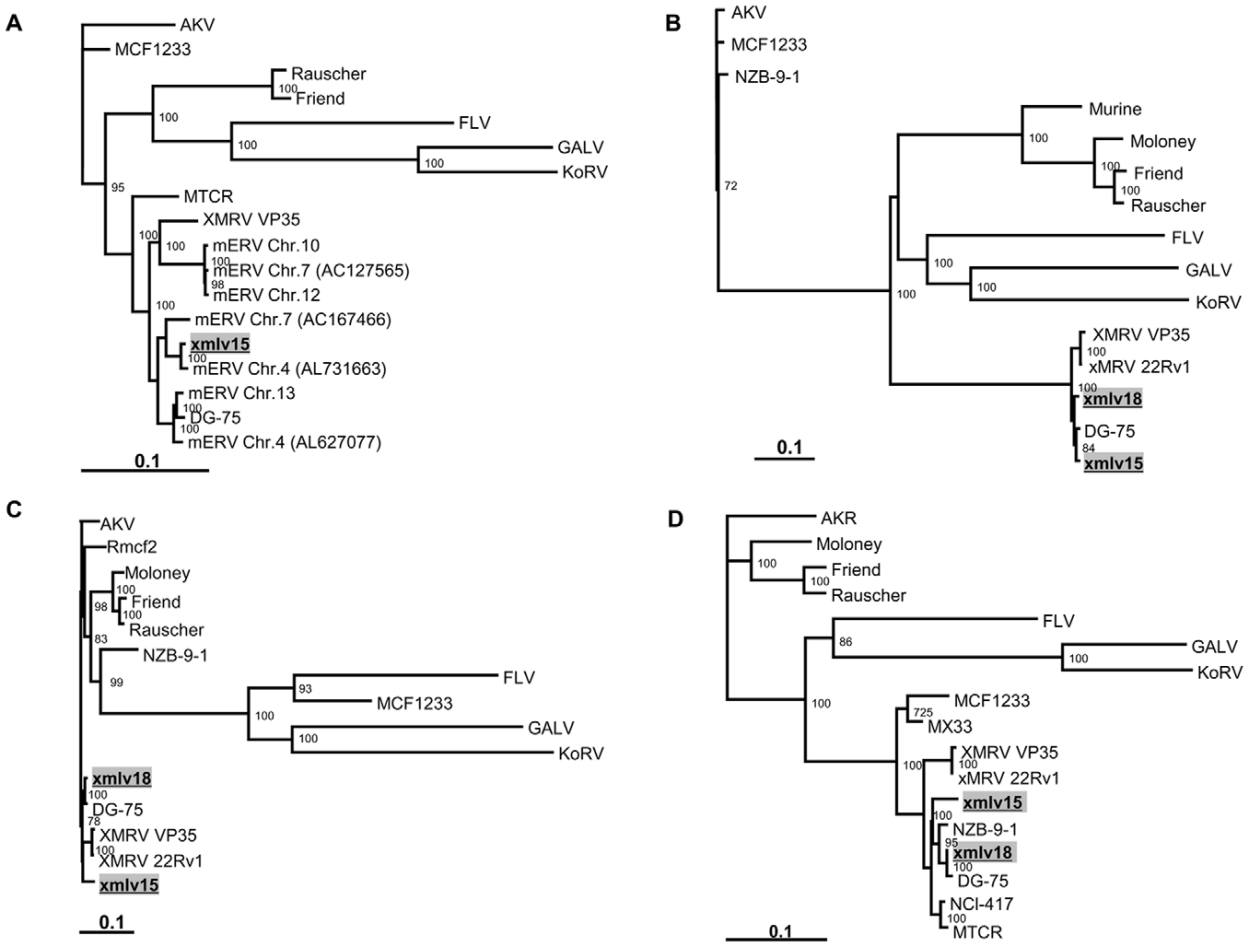
Phylogenetic analysis of the xmlvs. Phylogenetic trees were constructed based on (A) the complete genome sequences of the xmlv15 and (B–C) the predicted *gag*, *pro-pol* and *env* polyproteins of xmlvs. The full length nucleotide sequences and the deduced amino acid sequences of the xmlvs as well as the corresponding sequences from identified MuLVs were aligned using ClustalX. The resulting alignments were used to generate neighbor-joining trees. The values at the branch nodes represent the percentage of confidence in a specific branching. The sequences of the xmlvs (xmlv15 and xmlv18) are highlighted in gray. The sequences used in the analysis and in the construction are as follows: non-ecotropic proviruses (mERVs), AKV MuLV, Friend MuLV, Moloney MuLV, Rauscher MuLV, Feline leukemia virus, Gibbon ape leukemia virus, Koala retrovirus, DG-75, MTCR, MuLV MCF 1233, XMRV VP35, MuLV NCI-417, MuLV NZB-9-1, XMRV 22Rv1, MX33 and Rmf2.

**Figure 3:**
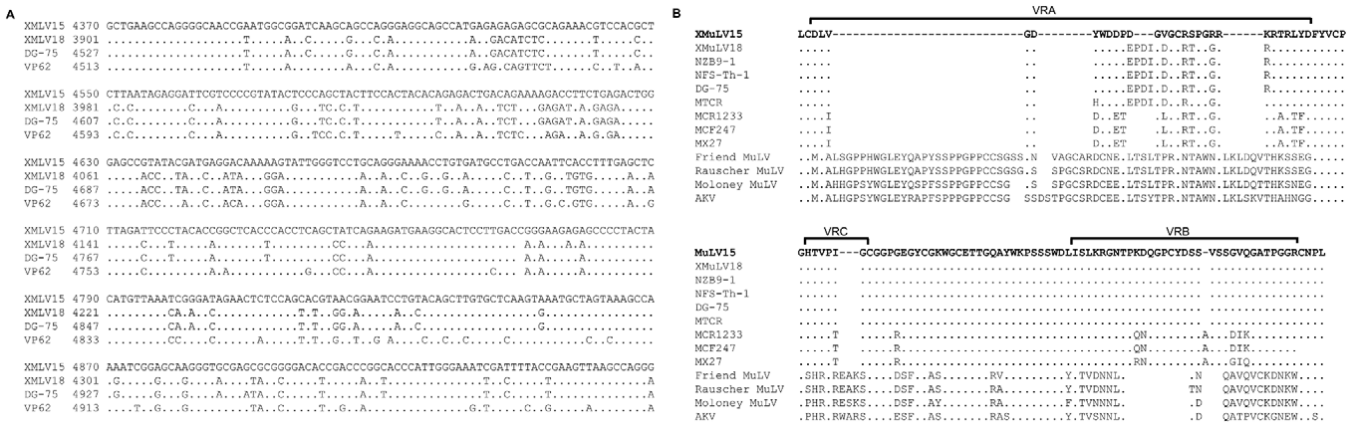
Multiple sequence alignment of xmlvs. (A) Sequence comparison of *pol* gene regions of xmlvs was performed with the corresponding regions of other X-MuLV isolates, DG-75 MuLV and XMRV VP62. (B) Multiple sequence alignment of the deduced amino acid sequences of xmlvs and related MuLVs spanning SU glycoprotein VRA, VRB and VRC, which were characterized by important regions for cellular tropism. This analysis employed the *env* protein sequences from the following viruses; AKV, Friend MuLV, Moloney MuLV, Rauscher MuLV, prototype polytropic clone MX27, DG-75, MTCR, MuLV MCF247, MuLV MCF 1233, MuLV NFS-Th-1 and MuLV NZB-9-1. The sequences were aligned using ClustalX. The dots indicate residues identical to those from xmlv15, and deleted residues appear as spaces.

The *Gag-pro-pol* region of the two xmlvs consisted of 5,202 nt with the potential to encode a *gag-pro-pol* polyprotein of 1,733 amino acids, and there were 235 nucleotide differences and 44 amino acid differences detected between xmlv15 and xmlv18. The coding domains found in the genomes of all known replication competent retroviruses encodes for polyproteins (*gag*, *gag-pro* or *gag-pro-pol*) whose cleavage products are the major functional proteins of the virus. Therefore, we analyzed the evolutionary relationships of each of the proteins encoded by the three xmlv genes (*gag*, *pol* and *env*) with other MuLV strains (Fig. 2B–D). The xmlv protein sequences were aligned with the corresponding sequences obtained from exogenous and endogenous retroviruses. Similar to the results of whole genome comparison, the entire *gag* sequences of the two xmlvs and DG-75 MuLV were found to be closely related, and xmlv15 in particular clustered with DG-75 MuLV to form distinct branches of a common phylogenetic node. In this region, there are 19 nucleotide differences between xmlv15 and DG-75 MuLV leading to 5 amino acid changes. Subsequently, we constructed a phylogenetic tree of the *pol* region, which encodes the longest retroviral polyprotein and is highly conserved because it encodes the viral enzymes, such as the viral protease, the reverse transcriptase and RNaseH proteins. In this region, 235 nucleotide differences and 36 amino acid differences were found between the two xmlvs, whereas this region of the xmlv18 sequence was very similar to that of DG-75 MuLV, showing 36 nucleotide differences resulting in 7 amino acid changes. Therefore, the *pol* region of xmlv18 was segregated together with DG-75 MuLV into distinct branches of a common phylogenetic node. As shown in Fig. 3A, we detected a higher degree of nucleotide variation in the terminal region of the xmlv15 *pol* gene compared to the other expressed X-MuLVs but one different nucleotide was found in this region when xmlve15 was compared with xenotropic provirus on *M. musculus* Chromosome 4.

### Analysis of the xmlv *Env* Region

The putative mRNA-coding region of the *env* gene from the xmlvs consisted of 1,935 nucleotides, and the *env* protein sequence of the xmlvs is 644 amino acids long. A pair-wise alignment indicated that xmlv15 and xmlv18 differ from each other by 57 nucleotides, leading to 28 amino acid changes. Phylogenetic analysis and comparison of amino acid sequences of the xmlv *env* proteins with other representative MuLV strains showed that the xmlv *env* regions exhibited the highest similarity with the *env* proteins of DG-75 MuLV and NZB-9-1 (Fig. 2C). Similar to other MuLV genomes, xmlv15 contained conserved splice donor (AGGTAAG, position 203) and acceptor (CACTTACAG, position 5436) sites used for the generation of the envelope. Multiple sequence alignment of the xmlv *env* region and the corresponding protein sequences of exogenous and endogenous MuLVs showed that xmlvs share the highest amino acid identity with xenotropic envelopes from NZB-9-1, NFS-Th-1 and DG-75 MuLVs. In spite of the high similarity detected, as shown in Fig. 3B, several amino acid substitutions unique to xmlv15 were present in variable region A (VRA) that are distinct from xmlv18 and other X-MuLVs.

### Analysis of xmlvs Expression

**Figure 4:**
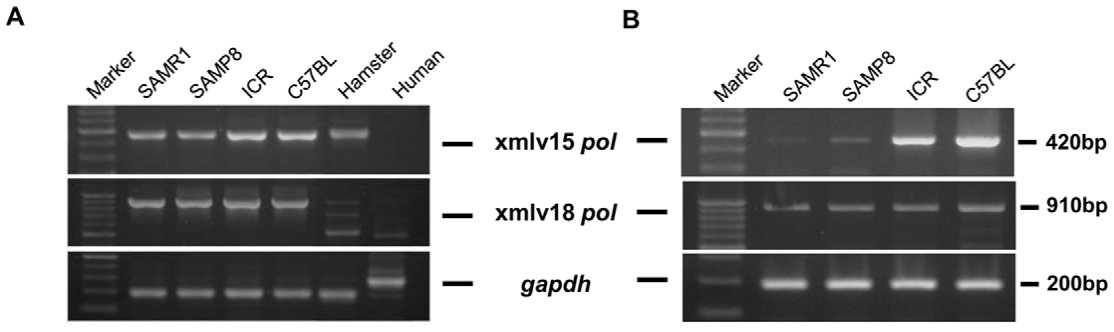
Detection of the xmlv proviruses. (A) PCR results screening of the genomic DNA of SAM, C57BL and ICR mouse strains, hamsters and humans. (B) RT-PCR analysis of xmlvs in the brains of various mouse strains, including SAMP8, SAMR1, C57BL and ICR.

To investigate the presence of the newly identified xmlvs in mice and other species, we amplified the *pol* regions of the xmlv15 and xmlv18-related sequences in the genomes of various mouse strains, hamsters and humans using primers for xmlv15 and xmlv18. As shown in Fig. 4, xmlvs-related sequences were detected not only in the SAM strain genome, but also in the common strains of laboratory mice, C57BL and ICR. In contrast, there were no xmlvs found in the hamster or human genome because the nucleotide sequences of PCR band that was found in the hamster species was not matched with any MuLVs. In conjunction with these results, xmlv15 and xmlv18-related mRNA could be detected in the examined mouse strains by RT-PCR analysis, but interestingly, the expression levels of xmlv15-related sequences were different between mouse strains. The xmlv15-related sequences were more highly expressed in C57BL and ICR mice than in the SAM mouse strains, whereas xmlv18-related sequences were expressed at similar levels in all of the mouse strains.

## Discussion

Since the MuLVs were first discovered in 1951 [20], various MuLV sequences have been isolated and investigated from various laboratory and wild mouse strains. As a result, many MuLV sequences have been identified *in vivo* and *in vitro* in mammals. However, most of these sequences were only partial sequences of MuLVs, such as those of *gag* or *env* regions. To clearly understand the characteristics and the function of each type of MuLV, further research and construction of databases including full genome sequences are necessary.

In the present study, we cloned and identified new X-MuLVs from the chromosomal DNA of a SAM strain. The xmlv15 was completely sequenced and was found to have a full-length viral genome of 8,176 bases. The xmlv18 was confirmed to have viral ORF region consisting of 7,077 bases. Through a comparative nucleotide analysis, we confirmed that the sequences of both xmlv15 and xmlv18 are clearly distinct from all known genomes of MuLVs even though they are very closely related to previously reported endogenous X-MuLVs that were found in C57BL/6J mice [21]. Since X-MuLV was originally isolated from the NZB mouse strain by Levy and Pincus in the 1970s [22], subsequent studies have reported that infectious X-MuLVs are found in the genomes of various inbred strains of laboratory mice [23], [24]. Our results showed that xmlv 15 and xmlv18-related sequences were not only present in the genomes of common laboratory mouse strains but also transcribed in their brains as well as in SAM strains. Interestingly, the expression levels of xmlv15-related sequences, not xmlv18-related sequences, are significantly different in the brains of C57BL and ICR mice compared with the expression levels in the brains of the SAM mice. Although similar sequences of endogenous X-MuLV could detected by our primers, the expression of xmlvs-related endogenous X-MuLVs have not been investigated in the previous study. The distribution of and ability to produce active X-MuLVs proviruses in these inbred mouse strains differ because of the presence of regulatory genes. The best characterized strains of inbred mice with a high-virus-expressing phenotype are F/St and NZB, which produce high titers of X-MuLV from an early stage [25], [26]. In other inbred strains, including C57BL and BALB/c, infectious X-MuLVs are rarely produced, but they can be activated by chemical and immunological stimulators [27]–[31]. One of four active X-MuLVs that can produce virus in laboratory mice, *Bxv1*, has been detected in one-third of the common strains of inbred mice [32]. In spite of the presence of the *Bxv1* provirus in the genome of C57BL mice [21], the expression levels of *Bxv1* are low on this mouse strain and in some other inbred mice. The expression levels of xmlv18-related sequences in all of the investigated mouse strains were consistent with the results of previous studies. However, the expression levels of xmlv15-related sequences in the brains of C57BL and ICR mice were relatively high. This finding suggested that there are uninvestigated endogenous X-MuLVs that have high transcriptional activity in the genomes of common inbred mice without any stimulators. X-MuLVs can infect cells of other species such as human and rabbit using the XPR1 receptor for cellular entry [33]. Horizontal transmission of MuLVs in mice has been reported [34], and mice and rats distributed worldwide can carry disease-causing agents that can infect humans and livestock [35]. Furthermore, previously published studies reported that new XMRVs are detected in humans [36]–[38], and that XPR1 is required for XMRV infection [39]. Overexpression of xmlv15-related sequences might represent a risk for laboratory workers who perform research on animals. In this regard, the investigation of endogenous X-MuLVs in individuals who usually handle laboratory mouse strains is important. We performed assays to detect xmlvs-related sequences using specific primers in the peripheral blood from 20 laboratory workers at the DNA and RNA levels. Fortunately, none of workers was positive for the newly identified xmlvs (data not shown). The transcriptionally active X-MuLVs may also influence the results of experimental infections of inbred mice with disease causing agents, such as viruses and peptides. Indeed, the levels of the X-MuLV expression in addition to the types of E- and P-MuLV were found to be increased in the brains of scrapie-infected SAM stains, which might affect the progression of scrapie pathogenesis in the SAM mice [40]. The association of the etiological agent of XMRV with human diseases is a highly controversial topic because convincing evidence demonstrating the possibility of contamination of mouse DNA in XMRV-positive samples have been reported [41]. Even if XMRV or related gammaretroviruses have not yet infected in humans, the risk of such infections occurring remains because X-MuLV receptor, Xpr1, is widely expressed in human tissue [42]. Since xmlvs have different transcriptional activity, more examinations will be needed to find the comprise characters of each xmlv. Therefore, we are planning to make construct of xmlvs for transfection study and to screen of xmlvs and xmlvs-related sequences activity before and after treatment of immune triggers in the organs of various mice including AKR mice.

In conclusion, we identified and characterized new X-MuLVs from the inbred mouse strain, SAM. We also found that the obtained X-MuLVs were proviral components of the genomes of other laboratory mice, and that considerable expression levels of xmlv15-related sequences were detected in the brains of C57BL and ICR mice without any stimulators. The findings of the present study will aid in building the database of complete genomes of X-MuLVs and understanding the roles of MuLVs under normal and/or pathogenic conditions.
